# Certain, but Not All, Tetraether Lipids from the Thermoacidophilic Archaeon *Sulfolobus acidocaldarius* Can Form Black Lipid Membranes with Remarkable Stability and Exhibiting Mthk Channel Activity with Unusually High Ca^2+^ Sensitivity

**DOI:** 10.3390/ijms222312941

**Published:** 2021-11-30

**Authors:** Alexander Bonanno, Parkson Lee-Gau Chong

**Affiliations:** Department of Medical Genetics and Molecular Biochemistry, Lewis Katz School of Medicine, Temple University, Philadelphia, PA 19140, USA; abonanno1116@gmail.com

**Keywords:** thermoacidophilic archaea, MthK, Ca^+2^-activated K^+^ channel, black lipid membrane, archaea tetraether lipids, polar lipid fraction E, membrane stability, channel activity

## Abstract

Bipolar tetraether lipids (BTL) have been long thought to play a critical role in allowing thermoacidophiles to thrive under extreme conditions. In the present study, we demonstrated that not all BTLs from the thermoacidophilic archaeon *Sulfolobus acidocaldarius* exhibit the same membrane behaviors. We found that free-standing planar membranes (i.e., black lipid membranes, BLM) made of the polar lipid fraction E (PLFE) isolated from *S.* *acidocaldarius* formed over a pinhole on a cellulose acetate partition in a dual-chamber Teflon device exhibited remarkable stability showing a virtually constant capacitance (~28 pF) for at least 11 days. PLFE contains exclusively tetraethers. The dominating hydrophobic core of PLFE lipids is glycerol dialky calditol tetraether (GDNT, ~90%), whereas glycerol dialkyl glycerol tetraether (GDGT) is a minor component (~10%). In sharp contrast, BLM made of BTL extracted from microvesicles (Sa-MVs) released from the same cells exhibited a capacitance between 36 and 39 pF lasting for only 8 h before membrane dielectric breakdown. Lipids in Sa-MVs are also exclusively tetraethers; however, the dominating lipid species in Sa-MVs is GDGT (>99%), not GDNT. The remarkable stability of BLM_PLFE_ can be attributed to strong PLFE–PLFE and PLFE–substrate interactions. In addition, we compare voltage-dependent channel activity of calcium-gated potassium channels (MthK) in BLM_PLFE_ to values recorded in BLM_Sa-MV_. MthK is an ion channel isolated from a methanogenic that has been extensively characterized in diester lipid membranes and has been used as a model for calcium-gated potassium channels. We found that MthK can insert into BLM_PLFE_ and exhibit channel activity, but not in BLM_Sa-MV_. Additionally, the opening/closing of the MthK in BLM_PLFE_ is detectable at calcium concentrations as low as 0.1 mM; conversely, in diester lipid membranes at such a low calcium concentration, no MthK channel activity is detectable. The differential effect of membrane stability and MthK channel activity between BLM_PLFE_ and BLM_Sa-MV_ may be attributed to their lipid structural differences and thus their abilities to interact with the substrate and membrane protein. Since Sa-MVs that bud off from the plasma membrane are exclusively tetraether lipids but do not contain the main tetraether lipid component GDNT of the plasma membrane, domain segregation must occur in *S. acidocaldarius*. The implication of this study is that lipid domain formation is existent and functionally essential in all kinds of cells, but domain formation may be even more prevalent and pronounced in hyperthermophiles, as strong domain formation with distinct membrane behaviors is necessary to counteract randomization due to high growth temperatures while BTL in general make archaea cell membranes stable in high temperature and low pH environments whereas different BTL domains play different functional roles.

## 1. Introduction

Bipolar tetraether lipids (BTL) have been long thought to play a pivotal role in allowing thermoacidophiles to thrive under extreme conditions such as high temperatures and low pH [1,2]. In the present study, using free-standing planar lipid membranes (also called black lipid membranes, BLM) as a model system, we attempt to address this question: Do all BTLs from the same thermoacidophilic archaeon exhibit the same (or similar) membrane behaviors and properties?

BLM over micro- or nano-pores in a solid thin film are useful tools for membrane biophysics studies [3] and excellent platforms for technological applications [4]. However, instability of the BLM made of conventional diester lipids has been a major drawback in these applications [5]. To remedy this problem, tough materials such as archaea tetraether lipids (reviewed in [6]), triblock copolymers [7,8] and polymerized lipids [3] have been used. Since the structure of lipids can affect biological activities of membrane-bound proteins [9], it is important to note that triblock copolymers and polymerized lipids are structurally very different from naturally occurring lipids, which could lead to unnatural responses from a biological sample. For instance, lipid polymerization reduces membrane fluidity, and the thickness of triblock copolymer membranes (typically 10–11 nm [10,11]) is much larger than that of eukaryotic cellular membranes (3.5–4.3 nm [12]). Both membrane thickness and fluidity can affect membrane protein behaviors [13,14]. Although biochemical activities can be detected when proteins insert into triblock copolymer or polymerized lipid membranes (reviewed in [15]), it is not clear to what extent the detected activities resemble those seen in membranes made of naturally occurring lipids.

**Figure 1:**
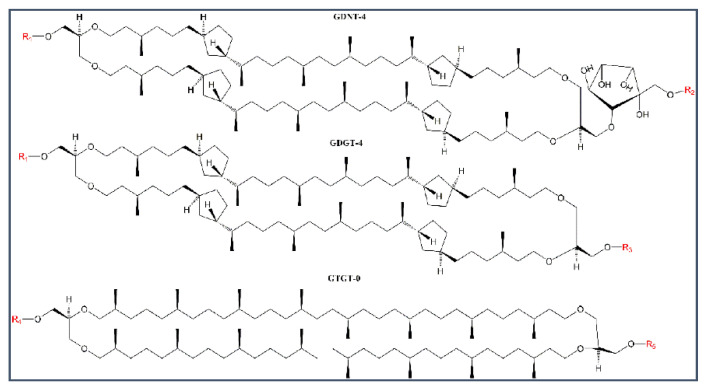
Illustration of the structures of archaea bipolar tetraether lipids (BTL). The major components for the hydrophobic core (in black) of archaea BTL include GDNT, GDGT, and glycerol trialkyl glycerol tetraether (GTGT) [33]. Microvesicles released from *Sulfolobus acidocaldarius* (Sa-MV) contain only GDGT and GTGT, lacking GDNT [32]. R1-R5 (in red) are the hydrophilic headgroups. For PLFE isolated from *S. acidocaldarius*, R1 = phospho-*myo*-inositol, R2 = β-d-glucopyranose, and R3 = β-d-galactopyranosyl-β-d-glucopyranose, and no GTGT was reported to be present [29]. The chemical structures of the hydrophilic head groups in Sa-MV lipids are not known [32]. The number after the abbreviations indicates the number of cyclopentane rings per molecule. In the cases of GDNT and GDGT isolated from *S. acidocaldarius*, this number can vary from 0–8 (reproduced from [34]).

BTL are the major lipid components in crenarchaeota, which are abundant not only in extreme environments such as hot springs and volcanic areas, but also in non-extreme environments such as soil and open sea [16,17]. BTL, along with diether lipids, are also found in methanogenic archaea [18], which are present in extreme environments, animals, and the human body [19]. BTL are macrocyclic or semi-macrocyclic compounds, containing branched methyl groups and cyclopentane or cyclohexane rings in the hydrocarbon chains, which are saturated and ether-linked to various polar headgroups such as phosphate and sugar moieties (illustrated in Figure 1) [20,21,22]. These structural features make BTL chemically more stable than diester lipids and yield vesicular membranes much more resistant against chemical, physical, and mechanical stressors than conventional diester liposomes [6,23].

Earlier studies showed that archaeal BTL can form free-standing planar membranes (BLM_BTL_) over micro-pores (0.6–1.2 mm) in a Teflon partition and stay stable for 5–6 h [24,25]. However, this membrane lifetime is not particularly remarkable compared to BLM from conventional diester lipids. Previous studies also showed that not all BTL can form stable BLM. For BTL isolated from the thermoacidophilic archaeon *Sulfolobus solfataricus*, the glycerol dialky calditol tetraether (GDNT), not the glycerol dialkyl glycerol tetraether (GDGT), component was able to form stable BLM [24]. Additionally, pre-treatment of the torus with lipids and the use of a particular organic solvent to make lipid stock solutions appear to be critical for formation of stable BLM_BTL_ [24,25]. For instance, applying a small amount of diphytanoylphosphatidylcholine (DPhPC) (a diester lipid with branched methyl groups) to the rim of the Teflon pores to form the torus led to stable BLM from the main tetraether glycophospholipids (MPL) isolated from the archaeon *Thermoplasma acidophilum*, while a higher amount of DPhPC applied to the torus yielded instable BLM_MPL_. In another example, applying a high amount of MPL in n-decane to the torus made stable BLM_MPL_, whereas using hexane as the MPL solvent yielded instable BLM_MPL_ [25]. Additionally, the thickness of BLM_BTL_ formed from n-decane was shorter than that from chloroform [26]. These results are interesting but puzzling; more studies are required in order to understand the factors contributing to the stability of BLM_BTL_. Furthermore, it has been shown that small peptides or ionophores, such as nonactin, valinomycin, and gramicidin, can insert into BLM_BTL_ and exhibit membrane conductance [24,25]; however, little is known about how larger membrane proteins behave in BLM_BTL_.

Our recent studies showed that certain BLM_BTL_ not only are more stable than BLM made of diester lipids (e.g., 1-palmitoyl-2-oleoyl-*sn*-glycero-3-phosphocholine, POPC, Figure S1) or triblock copolymers (e.g., poly(2-methyloxazoline)-block-poly(dimethylsiloxane)-block-poly(2-methyloxazoline), PMOXA-PDMS-PMOXA, Figure S1), but also exhibit unusual dielectric properties [27,28]. The polar lipid fraction E (PLFE) isolated from the thermoacidophilic archaeon *Sulfolobus acidocaldarius* contains exclusively BTLs [29]. We demonstrated that PLFE can form stable BLM on micro-pores (0.02–0.2 mm) of polydimethylsiloxane (PDMS) thin films embedded in a printed circuit board-based fluidics or in a glass/silicon microchip, exhibiting a constant electrical impedance for at least 55 h at 11–39 °C [27,28]. In contrast, BLM made of the diester lipid POPC (BLM_POPC_), prepared by the same procedures and on the same device, lasted only for 2 h before dielectric breakdown. We also found that although BLM made of the triblock copolymer (PMOXA-PDMS-PMOXA) can last as long as BLM_PLFE_, the impedance was not as stable, showing a significant drift in the beginning hours [28]. These two recent studies [27,28] indicate that the use of PLFE can greatly reduce the instability problem of BLM while avoiding the use of synthetic polymers, many of which are difficult to synthesize, costly, and not environmentally friendly. In addition, compared to BLM_POPC_ and BLM_PMOXA-PDMS-PMOXA_, BLM_PLFE_ exhibited unusual dielectric properties, showing an inductance component and a large membrane resistance [27]. The large resistance suggests that among the membranes examined, BLM_PLFE_ would be a better lipid matrix for studying channel proteins and transmembrane events. A stable BLM would lead to more robotic and reproducible measurements of the activities of membrane-bound proteins.

In the present study, we tested if the membrane-bound protein MthK can insert into BLM_PLFE_ and exhibit channel activity. MthK (~220 KDa) is a Ca^2+^-gated K channel, originating from the methanogenic archaea *Methanothermobacter thermautotrophicus*. MthK was chosen for this study because it has been extensively characterized and used as a model for calcium-gated potassium channels [30,31]. However, previous studies on MthK were confined to membranes composed of diester lipids [30,31]. Since the major membrane lipids in *M. thermautotrophicus* are mixtures of diethers and tetraethers [18], it is of physiological and biophysical interest to investigate how MthK behaves in archaeal BTL membranes. By using n-decane as the final solvent and by treating the torus with lipids before BLM formation over a micro pore on a thin film in a Teflon partitioning, we were able to make BLM_PLFE_ retaining a constant membrane capacitance for at least 11 days, which is unprecedentedly long for BLM made of naturally occurring lipids. We then compared MthK behaviors in BLM_PLFE_ (a GDNT-dominating planar membrane) with those in BLM made of lipids extracted from *S. acidocaldarius* microvesicles (Sa-MVs) (BTL_Sa-MV_, a GDGT-dominating planar membrane). PLFE isolated from *S. acidocaldarius* contains ~90% GDNT and ~10% GDGT [29], whereas lipids extracted from Sa-MVs are mainly GDGT, containing no GDNT [32]. Specifically, we examined MthK channel opening/closing in BLM_PLFE_ and BLM_Sa-MV_ at two different calcium concentrations. We were able to detect MthK’s channel opening/closing from BLM_PLFE_, but not from BLM_Sa-MV_. MthK’s channel opening/closing from BLM_PLFE_ occurs even at calcium concentrations as low as 0.1 mM. The unusually high sensitivity of MthK to calcium in BLM_PLFE_ is explained in terms of PLFE–PLFE and MthK–PLFE interactions.

## 2. Results and Discussions

### 2.1. Stability of BLM_PLFE_ Compared to Other BLMs

**Figure 2:**
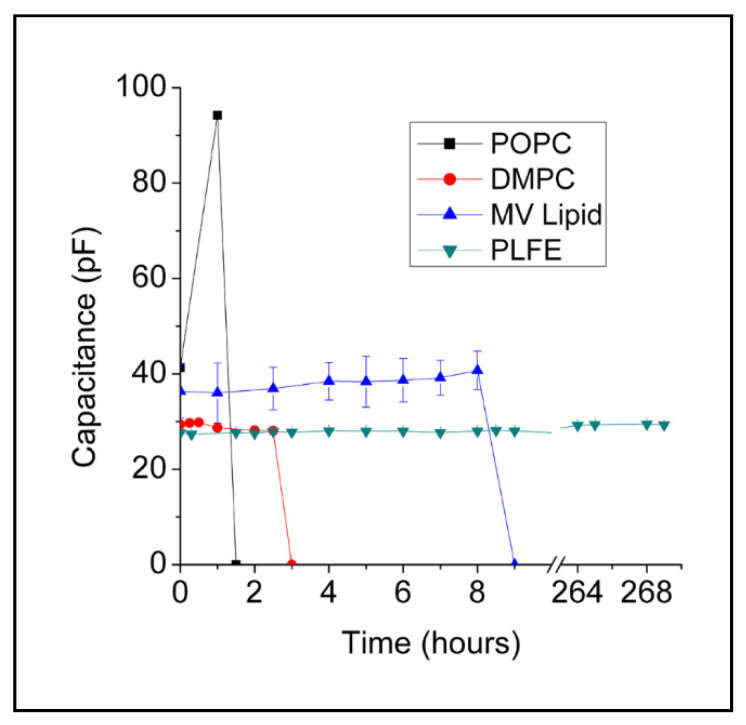
Time dependence of capacitance measured across various free-standing planar membranes. “MV lipid” denotes the membrane made of lipids (tetraethers) extracted from Sa-MVs. For the membranes made of POPC, DMPC and Sa-MV lipids, zero capacitance was detected at the end of the experiment, indicating membrane collapsing (electric breakdown). For PLFE planar membranes, within the instrument time allotted to us (11 days), there was no sign of capacitance drop. Capacitance was measured at ~22 °C.

Figure 2 shows that BLM_PLFE_ exhibits a constant capacitance (~28 pF) for at least 11 days. The experiment ended at Day 11, not because of membrane rupture or dielectric breakdown, but because of the limited instrument time. Being able to retain a constant membrane capacitance for such a long time (≥11 days) is unprecedented for BLM made of BTL (BLM_BTL_) or any other naturally occurring lipids, and is indicative of its remarkable stability. The previously reported BLM_BTL_ lasted only ~5–55 h [24,25,28]. Our present finding is significant because long-term stability is highly desirable to make BLM-based applications practically useful. The stability of BLM_PLFE_ reported in this study is comparable to that (4–24 days) of BLM made of synthetic polymers or made by photopolymerization of lipids [3,35,36], which, in contrast to archaea BTL, do not provide native lipid environments for membrane proteins and most likely are not environmentally friendly.

Like PLFE, Sa-MV lipids are exclusively tetraethers [32], however, BLM_Sa-MV_ are not as stable as BLM_PLFE_ (Figure 2). BLM_Sa-MV_ sustained a nearly constant capacitance (~36–39 pF) for only 8 h before electric breakdown (a zero capacitance). The hydrophobic cores of Sa-MV lipids are ~99.8% (by weight) GDGT and ~0.2% GTGT and contain no GDNT [32], whereas PLFE contains ~90% GDNT and ~10% GDGT [29] (Figure 1). Thus, the data of BLM_PLFE_ versus BLM_Sa-MV_ (Figure 2) seem to echo the previous finding that the GDNT, not the GDGT, component of the BTL forms stable BLM [24].

Figure 2 also shows that BLM_PLFE_ are much more stable than BLM made of conventional diester lipids (Figure 2). BLM_POPC_ had low stability, showing large fluctuations in capacitance before its collapse at 1.5 h. BLM_DMPC_ did not show capacitance fluctuations, but the membrane lifetime was also very short, i.e., ~3 h. A short lifetime is typical for free-standing planar membranes made of diester lipids [37,38].

#### 2.1.1. Explanations for the Differential BLM Stabilities

Understanding the origin of the remarkable stability of BLM_PLFE_ may lead to a strategy to optimize systematically BLM_BTL_ for an even longer lifetime and an even better membrane platform for applications. The molecular basis for the differential stability between BLM_Sa-MV_ and BLM_PLFE_ (Figure 2) must originate from the differences in lipid composition and lipid chemical structure. At present, a detailed structural comparison between PLFE and Sa-MV lipids is not possible because the chemical structures of the polar head groups on Sa-MV lipids are not known [32].

However, the remarkable stability of BLM_PLFE_ can be understood conceptually in terms of lipid–lipid and lipid–substrate interactions [39]. PLFE have phosphate and sugar moieties at both polar ends, which can form extensive hydrogen bond networks at the membrane surface [40,41]. In PLFE membranes, the macrocyclic dibiphytanyl chains containing cyclopentane rings and branched methyl groups (Figure 1) form a rigid and tightly packed hydrophobic core, with little membrane free volume fluctuations [23,42]. These structure features would provide strong lipid–lipid interactions and hinder membrane defect formation, thus generating great stability for BLM_PLFE_.

The interactions between PLFE lipids and the substrate (micro-pore and thin film from which the micro-pore was created) could be another major contributor to the remarkable stability of BLM_PLFE_. In our present study, the micro-pore was created by punching a pinhole on a piece of cellulose acetate film. Cellulose acetate is a polymer carrying multiple hydrogen bond acceptors and donors in each monomeric unit (Figure S1). PLFE have sugar moieties and phosphate groups at their polar headgroups (Figure 1). Conceivably, several hydrogen bonds can form between PLFE and the substrate, which would add stability to the planar membrane. This point can also be used to explain why BLM_PLFE_ made on cellulose acetate (the present study, ≥11 days) is more stable than BLM_PLFE_ made on PDMS ([28], ≥55 h = 2.3 days). PDMS has fewer hydrogen bond acceptors and donors per monomeric unit [28], compared to cellulose acetate.

BLM_Sa-MV_ is less stable compared to BLM_PLFE_ (Figure 2), which can be attributed to weaker lipid–substrate interactions, rather than weaker lipid–lipid interactions because membrane probe studies showed that liposomes made of Sa-MV lipids have tighter membrane (i.e., lipid–lipid) packing than PLFE liposomes [34].

Using the same concept of lipid–substrate interactions, we can explain why the lifetime of BLM made of the main glycophospholipids (MPL) isolated from the archaeon *Thermoplasma acidophilum* (~5–6 h) previously published by Stern et al. [25] was much shorter than the lifetime of BLM_PLFE_ reported in the present study (≥11 days). Firstly, the major component in MPL is a tetraether macrocyclic lipid containing cyclopentane rings and a head group of phosphoglycerol with a monosaccharide β-L-gulopyranose [43]. In comparison, PLFE have more sugar moieties than MPL in the polar head groups (Figure 1). In addition to phosphate, the head groups of GDNT component of PLFE have glucose, galactose, and *myo*-inositol. Thus, compared to MPL, PLFE lipids would have more intermolecular hydrogen bonds and thus stronger lipid–lipid interactions. Secondly, the MPL membrane used by Stern et al. [25] was formed over the micro-pore in a Teflon plate. The main component in Teflon is polytetrafluoroethylene (PTFE), which is hydrophobic. As a result, there is little interaction between the polar head groups of MPL and the substrate material (Teflon). In contrast, in the present study, micro-pores were created on a piece of cellulose acetate, which carried many functional groups (Figure S1) capable of forming multiple hydrogen bonds with PLFE lipids. Taken together, the stronger PLFE–PLFE and PLFE–substrate interactions explain why BLM_PLFE_ from the present study is much more stable than BLM_MPL_ reported by Stern et al. [25], despite the fact that both PLFE and MPL are bipolar tetraether lipids.

#### 2.1.2. Future Directions and Implications

More studies are required to evaluate further the contributing factors for the high stability of BLM_PLFE_. Upon proper optimization, it is possible to fabricate BLM_PLFE_ (or BLM made of BTL with structures similar to PLFE) with a membrane lifetime even longer than that currently reported (11 days). For example, we could optimize the critical factors in lipid–substrate interactions, which include the solvent used for painting lipids, the pretreatment of the torus with lipids, the shape of the micro-pore, and the pore size. In the study of Ren et al. [28], the solvent used was a mixture of chloroform/methanol/water, the edge of the pore was flat on both sides of the thin film, and there was no lipid pretreatment on the torus. In the present study, the final solvent used was n-decane, the edge of the pore was irregular and rough, and the torus was pre-treated with PLFE lipids. Stability of BLM is known to increase with decreasing the size of the pore over which the BLM is formed [44,45]. In the present study, the pore size was ~400 μm, which could be reduced to 20–100 μm in the future studies using photolithography or laser micromachining [28] to further increase stability of BLM_PLFE_. Such stable BLM_PLFE_ can be installed on microchips for various applications such as biosensing, drug screening, and artificial photosynthesis, to name a few [27,28].

Both PLFE and Sa-MVs originate from the plasma membrane of the archaea cell *S. acidocaldarius*. However, Sa-MVs and PLFE have very different lipid compositions. Sa-MVs are rich in GDGT with no GDNT whereas GDNT is the major component in PLFE, with GDGT as the minor component, as discussed earlier. This implies that lipid domain segregation occurs in the plasma membrane of *S. acidocaldarius*. Our present result (Figure 2) suggests that the lipid–substrate interactions could be very different in different lipid domains of the archaea and that, due to the stronger lipid–substrate interactions, the GDNT-dominating domains could play a more important role in archaea lipid biofilm formation than GDGT-dominating domains.

### 2.2. MthK in BLM_PLFE_

We used the archaea channel protein MthK as a model to test if the differential BLM stability described above affects membrane protein insertion and its biological activities. We incorporated MthK into liposomes made of *E.coli* lipids (see Materials and Methods) and introduced MthK-containing liposomes to the trans chamber of the BLM_PLFE_-containing Teflon partitioning device. The trans chamber was filled with HEPES buffer at pH 7.6, whereas the cis chamber contained HEPES buffer at a lower pH of 6.8 to suppress the channel activity (see Materials and Methods). MthK activity was monitored by measuring potassium flux through the membrane.

**Figure 3:**
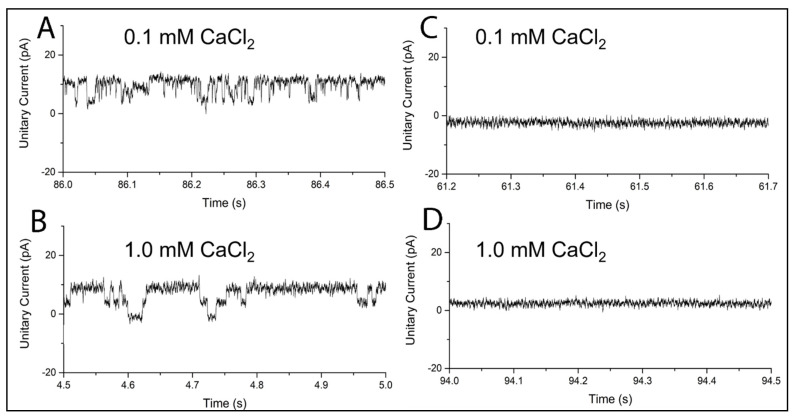
MthK channel activity in BLM_PLFE_ (**A**,**B**) or BLM_Sa-MV_ (**C**,**D**). Channel activity was measured at ~22 °C by potassium ion flux at two CaCl_2_ concentrations.

Figure 3A,B shows that, under an electric field of 100 mV, transmembrane current undergoes quantized jumps over time, which is indicative of channel opening. The open probability is higher at [Ca^2+^] = 1 mM than at [Ca^2+^] = 0.1 mM. These data show that the channel activity of MthK in BLM_PLFE_ is detectable in the presence of calcium chloride. To our knowledge, this is the first report of MthK channel activity in tetraether lipid membranes. Here we demonstrated that although PLFE lipid membranes are rigid and tightly packed [42], MthK can still insert into the PLFE membrane and exhibit channel activities.

It has been shown that membrane-bound proteins, including a leucine transport system, cytochrome-c oxidase, quinol oxidase, primary proton pumps, and isoprenylcysteine carboxyl methyltransferase, can insert into BTL-based liposomal membranes and retain their enzyme activities or transport functions [46,47,48,49,50,51,52]. Our current study shows that this property holds true in free-standing planar tetraether lipid membranes (BLM_BTL_).

Even more striking is the result of Ca^2+^ dependence of the MthK channel activity in BLM_PLFE_ (Figure 3A,B). At Ca^2+^ concentrations as low as 0.1 mM, the channel activity of MthK is still readily detectable in BLM_PLFE_ (Figure 3A). This result is surprising because very little channel activity (open probability ≈ zero) is detectable from MthK in diester lipid membranes. For example, MthK did not show channel activities in BLM made of 1-palmitoyl-2-oleoyl-*sn*-glycero-3-phosphoethanolamine (POPE) and 1-palmitoyl-2-oleoyl-*sn*-glycero-3-phosphoglycerol (POPG) (molar ratio 3/1) at [Ca^2+^] = 0.1–1.0 mM at pH 7.6 [31].

Ca^2+^ allosterically activates MthK. Specifically, Ca^2+^ binds to MthK domains called Regulator of K^+^ Conductance (RCK) at multiple sites. RCK domains form an octameric ring tethered to the pore of the channel. Binding of Ca^2+^ to the MthK RCK domains triggers a series of protein conformational changes, leading to channel opening and K^+^ conduction [31,53]. Compared to the results shown in [31], our current data indicate that Ca^2+^ activation of the MthK channel varies greatly with the type of lipid matrix. It is likely that calcium binding to MthK becomes much stronger when MthK is embedded in PLFE tetraether lipid membranes than in diester lipid membranes. As a result, less Ca^2+^ is needed in order to activate the channel in BLM_PLFE_.

Stronger Ca^2+^ binding may result from an unusual dynamic structure of MthK when it is in a PLFE lipid environment. A recent study showed that a channel protein can undertake a “rocking” motion in the membrane [54]. The “rocking” motion of MthK may be damped considerably in terms of amplitude and frequency when MthK is embedded in BLM_PLFE_. This is because some of MthK’s amino acid residues may have strong hydrogen bonding with neighboring PLFE polar headgroups, which are rich in sugar and phosphate moieties, and because the membrane-spanning domain of MthK is anchored in the rigid and tightly packed PLFE hydrophobic core. A more damped “rocking” motion of the channel protein in the membrane may increase the effective collision of Ca^2+^ to the MthK’s allosteric binding site, thus increasing the binding constant of Ca^2+^ to MthK. This proposition is supported by the finding that, when embedded in liposomal membranes made of *S. acidocaldarius* tetraether lipids, the electron dipole moment of bacteriochlorophyll *a* (BChl*a*) in the light-harvesting polypeptide (LH)/BChl*a* complex is more homogeneously and perpendicularly oriented with respect to the membrane surface [55].

While BLM_PLFE_ shows MthK channel opening/closing activities with high calcium sensitivity, no MthK channel activity was observed in BLM_Sa-MV_ at the same calcium concentrations (0.1–1.0 mM) employed and in the presence of the same amount of MthK-containing liposomes (Figure 3C,D), even though both PLFE and Sa-MV lipids are tetraethers derived from the same archaeon *S. acidocaldarius*. The inability to show channel activities in BLM_Sa-MV_ could be the result of the lack of incorporation of the MthK channel into the BLM or the lack of appropriate protein conformational changes required for channel activity due to a motionally more restricted environment in the polar head-group regions of Sa-MV lipid membranes, as indicated by higher values of red edge excitation shifts (REES) of Laurdan fluorescence in liposomes made of Sa-MV lipids compared to PLFE liposomes [34].

## 3. Materials and Methods

### 3.1. Isolation of PLFE and Sa-MV Lipids from the Archaeon S. acidocaldarius

*Sulfolobus acidocaldarius* cells (strain DSM639; ATCC, Rockville, MD, USA) were grown aerobically and heterotrophically at ~75 °C and pH ~2.7 [29]. Cell suspensions were centrifuged at 11,000× *g* for 20 min (rotor: Sorvall SLA-3000; centrifuge: Sorvall RC5B Plus, Waltham, MA, USA) and the pellets and supernatants were collected. The pellets were dried and PLFE lipids were isolated from the dry cells as previously published [56]. Sa-MVs were isolated from the cell supernatant using ultrafiltration (MWCO = 100 kDa, Millipore, Burlington, MA, USA) and ultracentrifugation (rotor: Ti867; centrifuge: Beckman XL-90, Indianapolis, IN, USA) on the cell supernatants as described [34,57]. Sa-MV lipids were extracted using a solvent mixture of chloroform, methanol and water (34.5:34.5:31, *v*/*v*/*v*) [57]. In addition to archaea tetraether lipids, we also used synthetic diester lipids 1-palmitoyl-2-oleoyl-*sn*-glycero-3-phosphocholine (POPC) and dimyristoyl-*sn*-glycero-3-phosphocholine (DMPC) obtained from Avanti Polar Lipids (Alabaster, AL, USA). A total of 400 μL of ~5 μM of lipid stock dissolved in chloroform (for POPC and DMPC) or chloroform:methanol:water (34.5:34.5:31, *v*/*v*/*v*) mixture (for PLFE and Sa-MV lipids) was dried under a slow nitrogen stream and re-suspended in 200 µL pentane. The pentane was then dried off under a slow nitrogen stream and the lipid was dissolved in an equal volume of n-decane.

### 3.2. Formation of BLM and Measurements of Membrane Stability

Black lipid membranes (BLM) were formed over a micro pore on a solid film in a dual-chamber Teflon device. Specifically, a pinhole (~400 µm in diameter) was poked through a clear cellulose acetate partition (transparency), and the pinhole was pre-treated with lipid dissolved in n-decane by using a glass wand and left to air dry for 10 min. The lower (trans) chamber was loaded with 10 mM 4-(2-hydroxyethyl)-1-piperazineethanesulfonic acid (HEPES) buffer containing 200 mM KCl and 0–1 mM CaCl_2_ at pH 7.6. After the addition of the trans well buffer, the pre-treated cellulose acetate partition was mounted onto the Teflon device separating the upper and lower chambers. The cis (top) chamber was then filled with 10 mM HEPES buffer containing 200 mM KCl at pH 6.8. Planar membranes formed spontaneously across the pinhole. If unsuccessful, the suspended lipid was agitated slightly by mechanical force of a small air bubble generated from the tip of a pipette. The chambers were connected to an Axopatch 200A amplifier (Molecular Devices, San Jose, CA, USA) via salt bridges containing 1 M KCl. Planar membrane formation was monitored electrically via continuous capacitance measurement at 22–24 °C.

### 3.3. Incorporation of MthK into BLM

*M. thermautotrophicus* MthK was expressed, purified, and reconstituted into proteoliposomes with the aid of the surfactant 3-((3-cholamidopropyl)dimethylammonio)-1-propanesulfonate (CHAPS, 34 mM) as described previously [31]. The isolated MthK was mixed with *E. coli* lipids (Avanti Polar Lipids, Alabaster, AL, USA) (50 μg protein/mg lipid) and bath sonicated for 5 min. Free MthK was removed from MthK-containing liposomes by using a Sephadex G-50 gel filtration column. MthK-containing liposomes were then added to the cis (top) chamber of the BLM-containing Teflon device where they were expected to spontaneously fuse with the planar membrane, depositing MthK therein.

### 3.4. Measurements of MthK Ion Channel Activities

Electrical signals (i.e., current) across the BLM were recorded in the presence of either 0.1 or 1.0 mM CaCl_2_ at 100 mV. The measured current change over time reflects ion flux across the membrane. Calcium was used to trigger MthK channel opening and closing. The solution on the cis side of the membrane (pH 6.8 and with no added Ca^2+^) suppressed the activity of MthK channels that were incorporated with their cytoplasmic face toward the cis chamber to nominal levels [31], resulting in the activation only of MthK channels with their cytoplasmic end facing the trans chamber.

## 4. Conclusions

This study shows that different BTLs that coexist in the plasma membrane of the thermoacidophile *S. acidocaldarius* can have very different membrane behaviors probably due to different BTL–BTL and BTL–substrate or BTL–protein interactions. The presence of various BTLs in thermoacidophiles likely leads to lipid domains in the plane of the cell membrane [58], each with a distinct membrane behavior. The GDGT-dominating domains, such as that associated with Sa-MV budding, may have slightly tighter lipid–lipid packing [34] but weaker interactions with solid substrate or membrane proteins (Figure 2 and Figure 3). The GDNT-dominating domains such as those formed by PLFE lipids may have strong BTL–substrate and BTL–protein interactions and can exhibit ion channel activities with high sensitivity. The implication of this study is that lipid domain formation is existent and functionally essential in all kinds of cells, but domain formation may be even more prevalent and pronounced in hyperthermophiles as strong domain formation with distinct membrane behaviors is necessary to counteract randomization due to high growth temperatures while BTLs in general make archaea cell membranes stable in high temperature and low pH environments [2] whereas different BTL domains play different functional roles.

The present study also shows that archaeal PLFE tetraether lipids are excellent biomaterials to make extraordinarily stable and yet biologically relevant free-standing planar membranes (BLM_PLFE_). BLM_PLFE_ can maintain a constant membrane capacitance for at least 11 days, accommodate the channel protein MthK, and display channel opening/closing activity at Ca^2+^ concentrations as low as 0.1 mM. The latter implies that, compared to conventional diester lipid membrane systems, MthK in BLM_PLFE_ has an unusual dynamic structure with stronger Ca^2+^ binding, which can more easily trigger MthK channel opening and closing. The extraordinary membrane stability and the high MthK sensitivity to calcium make BLM_PLFE_ an appealing platform for future studies of channel proteins and for developing new membrane protein-based technological applications.

## Data Availability

Data are contained within the article.

## Supplementary Materials

The following are available online at https://www.mdpi.com/article/10.3390/ijms222312941/s1.
