# Supplementary material for: Certain, but Not All, Tetraether Lipids from the Thermoacidophilic Archaeon Sulfolobus acidocaldarius Can Form Black Lipid Membranes with Remarkable Stability and Exhibiting Mthk Channel Activity with Unusually High Ca2+ Sensitivity
Source: Int J Mol Sci. 2021 Nov 30;22(23):12941. doi: 10.3390/ijms222312941 (PMC8657495; doi:10.3390/ijms222312941)
Supplement: Supplementary file 1 [file ijms-22-12941-s001.zip › ijms-1454412-supplementary.pdf]

Supplementary Materials:

Certain, but not all, tetraether lipids from the thermoacidophilic archaeon *Sulfolobus acidocaldarius* can form black lipid membranes with remarkable stability and exhibiting Mthk channel activity with unusually high Ca^{2+} sensitivity

Alexander Bonanno and Parkson Lee-Gau Chong*

Department of Medical Genetics and Molecular Biochemistry, Lewis Katz School of Medicine, Temple University, Philadelphia, PA 191040

*Corresponding author: Parkson Chong, email address: pchong02@temple.edu

Email addresses:
Alexander Bonanno: abonanno1116@gmail.com
Parkson Lee-Gau Chong: pchong02@temple.edu

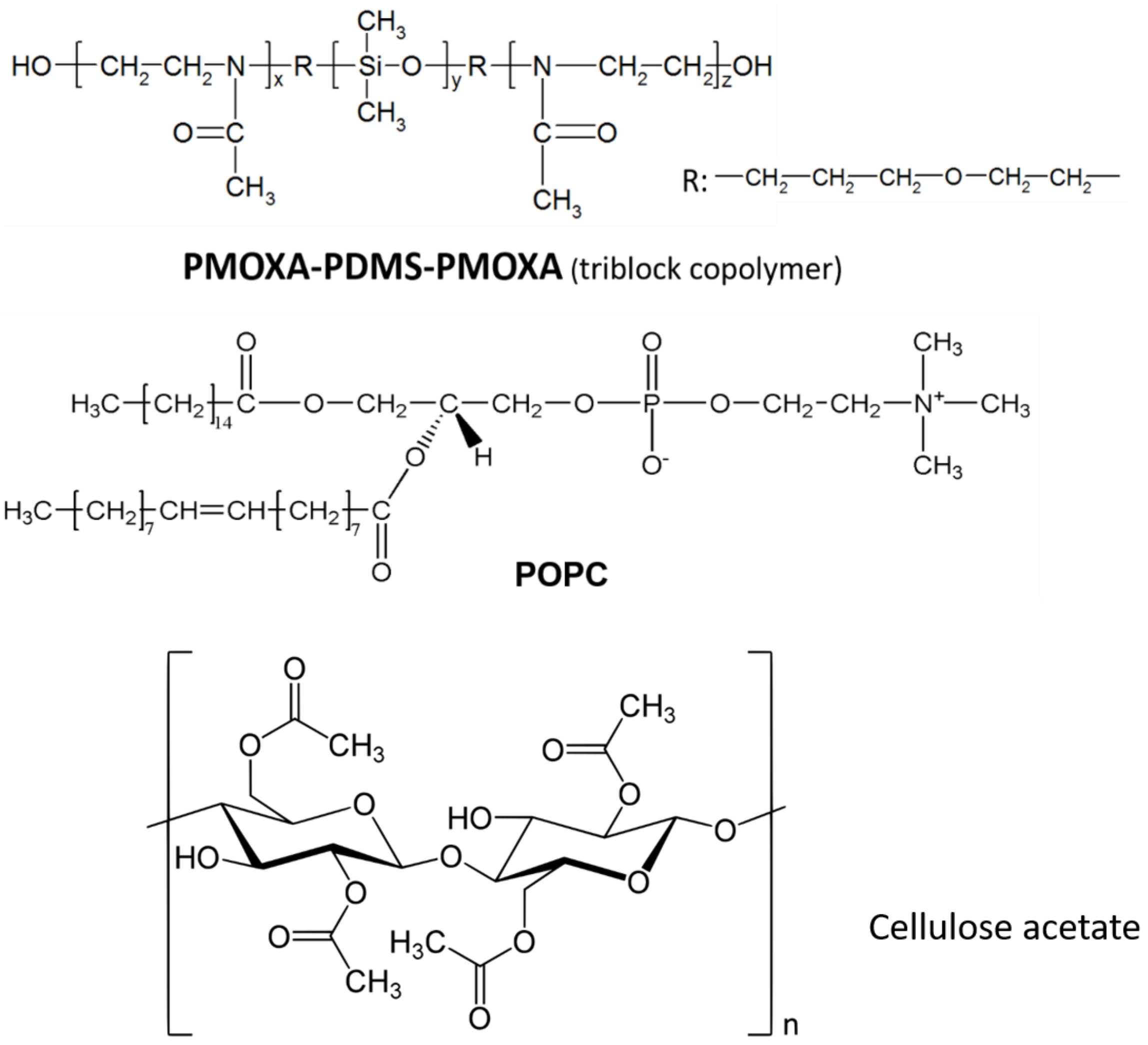

Figure S1. Chemical structures of the triblock copolymer PMOXA-PDMS-PMOXA, the diester lipid POPC, and the polymer cellulose acetate.
